# A Donders’ Like Law for Arm Movements: The Signal not the Noise

**DOI:** 10.3389/fnhum.2016.00136

**Published:** 2016-03-30

**Authors:** Steven Ewart, Stephanie M. Hynes, Warren G. Darling, Charles Capaday

**Affiliations:** ^1^Department of Health and Human Physiology, Motor Control Laboratories, University of IowaIowa City, IA, USA; ^2^Institute of Neurorehabilitation Engineering, Bernstein Focus Neurotechnology Göttingen, Bernstein Center for Computational Neuroscience, Universitätsmedizin Göttingen, Georg-August-UniversitätGöttingen, Germany

**Keywords:** Donders’ law, arm kinematic rules, motor cortex, arm movements, pointing movements, grasping movements

## Abstract

Experiments were done to determine whether the starting position of the arm influences its final configuration (posture) when pointing to, or grasping, targets located within the common workspace of the arm. Subjects were asked to point to, or grasp, each of six targets from five, or seven, widely spaced starting positions. We found that the variability (standard deviation) of the arm’s configuration, measured as the angle of inclination of the plane delimited by the arm and forearm, averaged about 4° for comfortable speed pointing movements and was only slightly higher for fast pointing movements. Comfortable speed reaches to grasp the targets were associated with slightly lower variability (3.5°) in final arm configuration. The average variability of repeated movements to a given target from a single start position (3.5°) was comparable to that of movements from different start positions to the same target (4.2°). A small difference in final arm inclination angle, averaged across all subjects and targets, of 3° was found between two pairs of starting positions. This small and possibly idiosyncratic effect is within the “noise” of final arm orientation variability for repeated movements (i.e., 3.5°). Thus, the variability of final posture is not for the most part due to different start positions, it is inherent to movement *per se*. Our results reconcile conflicting previous studies and are consistent with past works suggesting that a Donders’ like law is indeed largely upheld for unconstrained visually guided arm movements. In summary, considering movements within a typical work space, when the hand is moved voluntarily to a given spatial location the posture of the arm is nearly the same regardless of its starting position. Importantly, variability is inherent to the rule.

## Introduction

A complex multi-jointed system like the human arm is difficult to control. Inertial interaction forces between joints, changes of the moment of inertia with arm configuration, nonlinear gravitational force effects and the fact that many muscles span more than one joint must be taken into account to formulate movement commands (Hollerbach and Flash, 1982; Atkeson and Hollerbach, 1985; Cooke and Virji-Babul, 1995; Virji-Babul and Cooke, 1995). In addition to the dynamic factors there are also kinematic factors that must be considered. The human arm has, neglecting movements of the shoulder blade and assuming no motility of the hand itself, seven degrees of mechanical freedom (i.e., joint rotation axes). This is one more degree of freedom than necessary to arbitrarily orient and position the hand at a given spatial location. The joint axes that are involved in a given arm movement will determine the trajectory of the hand in space and its final posture. How then is a particular combination of joint rotational axes selected for a given movement? This problem is referred to as the degrees of freedom problem (Bernstein, 1967). An answer to the how question, in terms of neural mechanisms, would be greatly facilitated if there were a basic rule governing the selection process. Attempts to determine the existence of such a rule and more generally the control principles underlying movements of the arm have led to conflicting conclusions (Soechting et al., 1995; Desmurget and Prablanc, 1997; Gielen et al., 1997; Grea et al., 2000; Schot et al., 2010). Nonetheless, the search for such a rule is thought to be important because it can lead to well posed questions for investigating the central neural mechanisms involved in controlling arm movements.

For saccadic eye movements a simple rule was discovered by the 19th century physiologist Donders (1876). The rule, known as Donders’ law, states that for each gaze direction there exists a single corresponding eye orientation irrespective of how the eye was brought to this position (Alpern, 1969; Nakayama and Balliet, 1977; Tweed and Vilis, 1990). The rule also holds during smooth pursuit eye movements and normal movements of the head (Misslisch et al., 1994). Thus, only two variables, azimuth and elevation which define gaze direction, are needed to describe the orientation of the eye, or head. The third variable (orientation, or roll) is a function of the other two. Because the ocular globe and the head can move about three axes, Donders’ law is an example of dimensionality reduction, three potential degrees of kinematic freedom are effectively reduced to two. The functional significance of Donders’ law is yet to be determined. One possibility is that the constraint helps with visual perception, as originally proposed by Helmholtz, another is that it simplifies the required neural control signals (Quaia and Optican, 2003). To explain Donders’ law, Listing proposed that the eye makes what is in effect a single rotation from the primary reference position of the eye (roughly with the head erect and the eyes looking at the horizon) about a given axis (Alpern, 1969). All such axes of eye rotation lie on a plane, known as Listing’s plane which is tilted approximately 20° counterclockwise from the vertical when the head is held upright (Tweed et al., 1990). It is important to note that Donders’ law is a physiological finding, whereas Listing’s law is its most parsimonious geometric explanation (Alpern, 1969). Optokinetic (OKN) and vestibulo-ocular (VOR) reflex movements, however, do not follow Donders’ law. This is usually taken as evidence that Donders’ law has a neural origin. Nonetheless, the manner in which extraocular muscles attach to the orbital globe is thought to simplify the neural implementation of Donders’ law (Quaia and Optican, 2003).

An articulated body part which obeys a Donders’ like law would assume a particular end configuration, or posture, at a particular movement endpoint. Moreover, this configuration would be independent of the movement’s start position. In this operating mode, the CNS would move the arm, for example, to the particular end posture corresponding to the intended movement endpoint, regardless of the arm’s starting position. This provides a relatively simple solution to a complex problem and we have hypothesized that this is the *de facto* mode of motor cortex operation (Capaday et al., 2013). However, our hypothesis falls amidst seeming contradictions and consequent controversy, a point that has been made by others (e.g., see Medendorp et al., 2000). On the one hand, Hore et al. (1992) found that in the simple case of pointing with a fully outstretched arm, Donders’ law was indeed obeyed. The fully outstretched arm adopts a nearly similar orientation when pointing at a given location, regardless of the arm’s start position. Consequently, as with the eye, only azimuth (yaw) and elevation are needed to describe arm orientation in this task. This was later confirmed by Liebermann et al. (2006) and also for pointing with the hand when only wrist motion was allowed (Campolo et al., 2010). On the other hand, studies of the outstretched arm by other groups concluded that upper limb configurations in straight arm pointing do not obey Donders’ law (Miller et al., 1992; Gielen et al., 1997; Admiraal et al., 2004). Surprisingly, however, the data presented in these studies show that the law’s predictions are approximated to within a few degrees as the variations of arm orientation at a given target had a standard deviation of ~3–4°, equivalent to the values reported by Hore et al. (1992).

Pointing with a straight arm constrains movements to three degrees of freedom at the shoulder, far fewer than available for the arm as a whole. On this basis, the issue was re-examined by Soechting et al. (1995). They reported that for relatively unconstrained pointing movements the final orientation of the plane of the arm (a measure of arm posture) showed large variations at a given spatial location for different starting positions. Thus, according to these authors a Donders’ like law is not obeyed for unconstrained movements of the arm. By contrast, again, a Donders’ like law for final upper limb postures was obeyed to within a few degrees for unconstrained reaching movements from various starting positions to grasp spherical targets (Schot et al., 2010).

Given the unresolved status of the problem, we sought to determine whether in fact, accepting the variability inherent to physiological processes, a Donders’ like law holds in natural unconstrained arm movements. Specifically, we hypothesized that pointing movements made at comfortable speed starting from different locations to place the tip of the index finger over a target would exhibit low variability in arm posture at the endpoint. Fast pointing movements we expected would have larger variations because previous research showed that rapid head movements do not follow Donders’ law (Tweed and Vilis, 1992). Moreover, fast pointing movements exhibit greater variability of index fingertip position at the endpoint. They are less accurate (Fitts, 1954), their trajectory is more variable than that of comparable slow movements (Darling and Cooke, 1987), and their planning may additionally involve consideration of energy requirements to mitigate fatigue (Elliott et al., 2009). We also hypothesized that reaching to grasp and lift a cylinder at comfortable speeds from different starting locations would exhibit low variability in final arm posture, because the hand orientation required to grasp the object needs to be matched to it.

We reasoned that, if these different classes of arm movements all exhibit low variability of the arm’s posture at the endpoint, independent of its start position, this would demonstrate that the CNS does follow a simple Donders’ like rule to control such movements. In the event, this would strongly inform the search for central neural mechanisms of motor coordination.

## Materials and Methods

### Subjects and Ethical Approval

**Figure 1:**
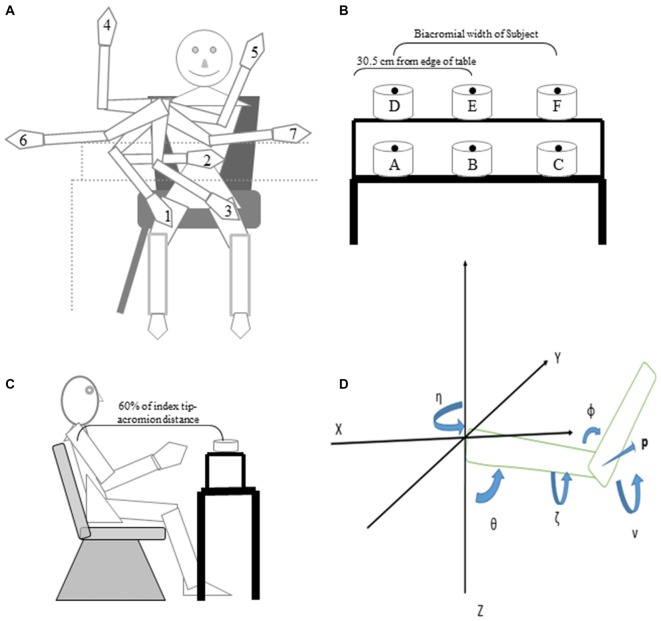
**Cartoon of starting arm positions, targets and the joint angles that define the arm orientation in space. (A)** Subjects pointed to or reached to grasp six targets from the start positions depicted in the cartoon. **(B)** Subject’s view of the table and locations of the six targets. **(C)** Side view of subject, table, shelf and one target. Note that the drawings are not to scale in terms of relative size of the subject, targets and table. **(D)** Starting from a position with the arm pointing forward (along the *X* axis), the yaw angle η represents a rotation of the arm about the vertical *Z* axis and defines the plane of elevation of the arm; the elevation angle θ represents a rotation about the medial-lateral axis of the arm after the yaw rotation; humeral rotation, ζ represents rotation about the long axis of the humerus. The vector, **p**, perpendicular to the plane of the arm, provides a concise description of the orientation of the upper limb in space. The angle of inclination ν is the acute angle between **p** and the horizontal plane and is a succinct measure of arm posture at given hand location.

Eight right handed subjects (5 males), 25.25 ± 4.37 (mean ± SD) years of age, with no history of neurological or muscular disorders participated in these experiments. Subjects were asked to point to, or grasp, six targets spaced widely within the arm’s workspace. Each of these targets was pointed to, or grasped, starting from five, or seven, widely different initial arm positions (Figure 1). The study was approved by the University of Iowa Institutional Review Board. All subjects signed informed consent documents before participating. The subjects were uninformed as to the purpose of the study. Right handedness was confirmed by the revised Edinburgh Handedness Inventory. Subjects received compensation for their participation.

### Experimental Setup

Each subject’s biacromial width and right arm length (from the distal end of the index finger to the acromion) was measured. This measurement allowed the experimenter to control for different sized subjects by adjusting target locations and the subject’s distance from the table where the targets were located. The subject sat naturally in front of a table, which was a distance of 60% of the arm’s length to the xiphoid process. Subjects were asked to remove any metal objects because such objects may interfere with the devices used to record arm position and motion.

Position and orientation of the upper limb segments was recorded using electromagnetic systems (Ascension Technologies minibird and trakstar systems, Burlington, VT, USA). Sensors were attached to the skin over the distal phalanx of the index finger, the styloid process of the ulna, the lateral epicondyle of the humerus, and the acromion process of the scapula. The sensors were secured to the skin with double-sided tape under the sensor and single-sided tape to attach the wires to the skin to minimize strain on the sensors, tangling of wires, and to ensure the subject had a full unhindered range of motion at the shoulder elbow, wrist, and finger joint. The transmitter rested on the left side of the table. Each sensor’s X, Y, Z, azimuth, elevation, and rotation in space were recorded at 74 Hz using Skill Technologies 6D Research software (minibird system) or at 200 Hz (trakstar system) using a custom MATLAB program (Mathworks, Natic, MA, USA) and then transferred to Datapac 2k2 (Run Technologies, Laguna Hills, CA, USA) for data analysis.

### Experimental Protocol

After the subjects were familiarized with the experimental setup, they were asked to perform two to four practice trials to various targets until they felt comfortable with the task. During this time, subjects were familiarized with the five or seven starting positions. Each of the six targets was a circular piece of tape, 3.5 cm in diameter, marking the middle of a cylinder, which was 5.1 cm in diameter, 4.4 cm in height and weighing 340 grams.

Once comfortable, subjects executed four blocks of trials in which they would start from one of five (subjects 1–4), or seven (subjects 5–8), widely spaced locations as shown in Figure 1A and described further below. They were instructed to position the index finger just above the center of a cylinder positioned at one of the six locations (Figure 1B) placed on a table (targets A, B, C) or a shelf positioned 19.7 cm above the table (Targets D, E, F). Targets B and E were placed directly in front of the midline at a distance equal to 60% of arm’s length (Figure 1C) from the xiphoid process (acromion to index tip) with target E located 19.7 cm directly above target B. Targets A and C were on the table and set a distance apart equal to the subject’s biacromial breadth. Targets D and F were on the shelf directly above targets A and C.

The first four subjects were tested with five hand starting locations (1–5 in Figure 1A). We then added two more starting hand locations (6, 7 in Figure 1A) to test a wider range of starting locations in four additional subjects. The starting positions were as shown in Figure 1A: (1) hand on right mid-thigh; (2) hand on the abdomen; (3) hand on the left mid-thigh; (4) arm abducted 90° from the torso, elbow flexed 90° such that the right hand was pointing upward; (5) arm horizontally adducted as far as was comfortable, elbow flexed 90° such that the right hand was pointing upwards; (6) arm abducted 90°, elbow extended so that the hand was pointing to the right; and (7) arm horizontally adducted the arm as far as was comfortable, elbow slightly flexed so that the right hand was pointing to the left.

There were two blocks of 30 trials (5 starting positions) or 42 trials (7 starting positions) in which the subject was instructed to reach at a comfortable speed and two blocks of 30 or 42 trials in which the subject was told to reach quickly. In both conditions subjects were told to finish the movement with the right index finger just above, not touching, the center of the target. These blocks were performed in a random order for each subject. Starting position of the hand and target locations were randomized within blocks as well. After these four blocks were completed the subject completed two blocks of 30 or 42 trials under the instruction to grasp the cylinder with the first three digits (thumb, index finger, and long finger) with the palm of the hand directly above the target (i.e., similar to the pronated hand posture for pointing), and then pause before carrying it two inches forward past a string attached to the table and then back to the initial target location. Between each two block set, subjects were given the opportunity to rest for 1–2 min as desired.

Subjects produced movements in an unconstrained three-dimensional environment for all movement conditions. Torso and wrist movements were unrestricted to allow maximum degrees of freedom in the upper limb. This freedom allowed for the greatest possible variability in final arm postures and more closely approximates movements in everyday environments than in many previous experiments that constrained trunk, wrist, and/or elbow movements (Cruse, 1986; Cruse and Bruwer, 1987; Uno et al., 1989; Straumann et al., 1991; Hore et al., 1992; Gielen et al., 1997; Medendorp et al., 2000; Papaxanthis et al., 2003; Admiraal et al., 2004; Kistemaker et al., 2010).

In each block of trials, the subject performed movements to a particular target from five (the first four subjects) or seven (the last four subjects) starting positions in random order before the target was relocated to a new position on the table or shelf. Verbal instruction was provided about the starting location before the movement; subjects were allowed time to put the arm in this position. Subjects were instructed to move by a verbal command “go” or “reach” given by the experimenter. The subject completed the movement and maintained their final position for a second, or two, until prompted by the experimenter to return their hand onto their lap.

### Data Reduction and Analysis

A low pass Butterworth filter was applied to the motion of each sensor with a cutoff of 10 Hz. Each movement was visually analyzed, using a display of index tip X, Y, and Z position and velocity vs. time to mark the index tip sensor’s initial and final positions. This process was done visually instead of using a velocity criterion for movement onset and termination due to small tremors associated with holding the arm in starting and ending positions. The starting position was marked a few milliseconds before movement was initiated and the movement was marked as complete when the hand position was constant. Movements that were not fully captured within the 5 second recording period were removed from the analysis. These missing data occurred in three subjects and ranged from 1 to 5 trials missing from among 14 trials for a particular target/condition.

Yaw, elevation and roll of the arm (humeral) and forearm segments (radius and ulna) were calculated as a series of ordered rotations about axes fixed to the segment from a standard upper limb configuration in which the arm was pointing straight forward from the subject. The plane of the upper limb and orientations of the arm (humerus) and forearm segments were defined from the XYZ positions of the acromion, humerus lateral epicondyle and styloid process of the ulna recorded from the electromagnetic sensors (Figure 1D). Referring to Figure 1D, the yaw angle η was defined as a rotation of the segment about the vertical axis with 0° being directly forward from the subject and 90° being directly to the left of the subject (positive *Y* axis). The second rotation was about a horizontal axis (medial to lateral in the standard configuration) and defined elevation θ of the segment, which was measured as the angle of the segment from the vertical *Z* axis with 0° indicating that the arm segment is pointing straight down and 90° corresponding to a horizontal segment. The third rotation, at the shoulder only, was about the long axis of the humerus to define arm roll ζ (internal/external rotation). The angle of inclination of the plane of the upper limb ν defines the configuration of the arm in three dimensions. This angle is derived from the vector **p**, the cross product of the arm and forearm vectors, which is perpendicular to the plane of the arm. Once this vector is calculated, its angle relative to the XY plane (Figure 1D) is computed by the equation *sinν = sinθsinζ*. Put simply, ν is the angle between the vector perpendicular to the plane defined by the arm and forearm and the horizontal XY plane, the angle being 0 if the arm plane is vertical.

This measure of arm posture is the same as that used by Soechting et al. (1995) and provides a simple measurement of upper limb configuration, because for a given index fingertip location the inclination angle will be constant if arm and forearm yaw and elevation angles are the same, assuming that the wrist and finger have similar contribution to the movements. Previous studies have reported that wrist movement produced minimal variation in end point posture of the arm and forearm compared to the shoulder (Miller et al., 1992; Wang, 1999; Schot et al., 2010). We stress that it is the variation of this endpoint posture measure that is of most interest. The orientation of the arm plane measure we used distills several variables into one. However, whilst expedient, our measure of arm posture is not related one to one with its actual posture. We have used it, as did Soechting et al. (1995), to obtain a measure of the variability (SD) of arm posture.

### Statistical Procedures

A 6 × 3 repeated measure ANOVA was used to test within subject effects of target locations (A-F) and conditions (comfortable reach, quick reach, and grasp) on mean final arm orientations (inclination angles of the plane of the upper limb) for movements from different starting positions to test whether mean orientations (over 5 or 7 different starting hand positions) differed for different targets and conditions. This analysis showed that mean final arm orientations did not differ for comfortable speed and quick reaching movements, but were different for reaches to grasp the targets (see “Results” Section). We therefore tested our main hypothesis concerning effects of hand starting position on mean final orientations of comfortable speed and quick reaching movements from the five starting positions common to all subjects using a 6 (targets) × 5 (hand starting positions) repeated measures ANOVA. We also used a 6 (targets) × 3 (conditions) repeated measures ANOVA to test whether variability of final arm orientations for the different targets differed for the different conditions. Mauchly’s test was used to check for sphericity and Huynh-Feldt corrections were applied when necessary to compute *p*-values based on adjusted degrees of freedom. *Post hoc* Tukey’s HSD tests were used to assess differences among individual targets and conditions if main or interaction effects were statistically significant.

## Results

**Figure 2:**
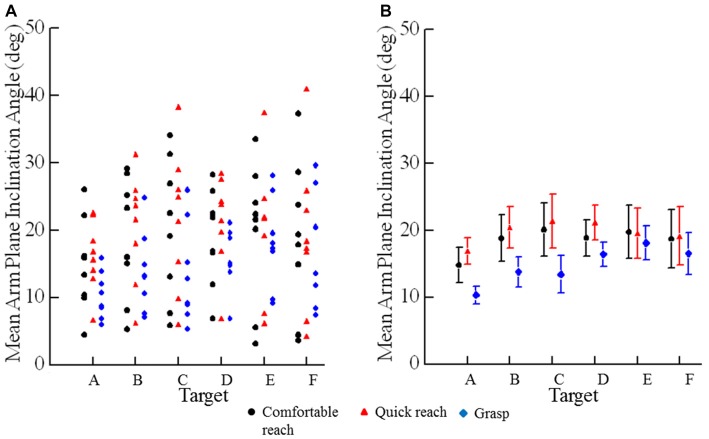
**Mean angles of inclination of the plane of the arm for individual subjects. (A)** Each plotted point is an individual subject’s mean angle of inclination to a specific target (A–F) under a specific condition (comfortable reach, quick reach, or grasp). **(B)** Each plotted point is the mean angle of inclination across all subjects to a specific target (A–F) under a specific condition (comfortable reach, quick reach, or grasp). Error bars are ±1 SE.

The mean angle of inclination of the arm at endpoint (Figure 2) depended on target location and reaching condition (condition × target interaction *F*_(10,70)_ = 4.46, *p* < 0.001). The mean angles were slightly lower for reaches to grasp than for comfortable and quick reaches to point at targets (Figure 2B). The mean inclination angles of the arm were similar for comfortable and quick reaches (*p* = 0.830) as can be seen in Figure 2B. Each subject had their own characteristic arm configuration at a given target, as can be inferred from the vertical scatter of the data points (Figure 2A). Summarizing, final arm configurations when pointing at targets were not affected by movement speed. However, reaches to grasp the same targets used slightly different final arm configurations, as would be expected (Figure 2B).

**Figure 3:**
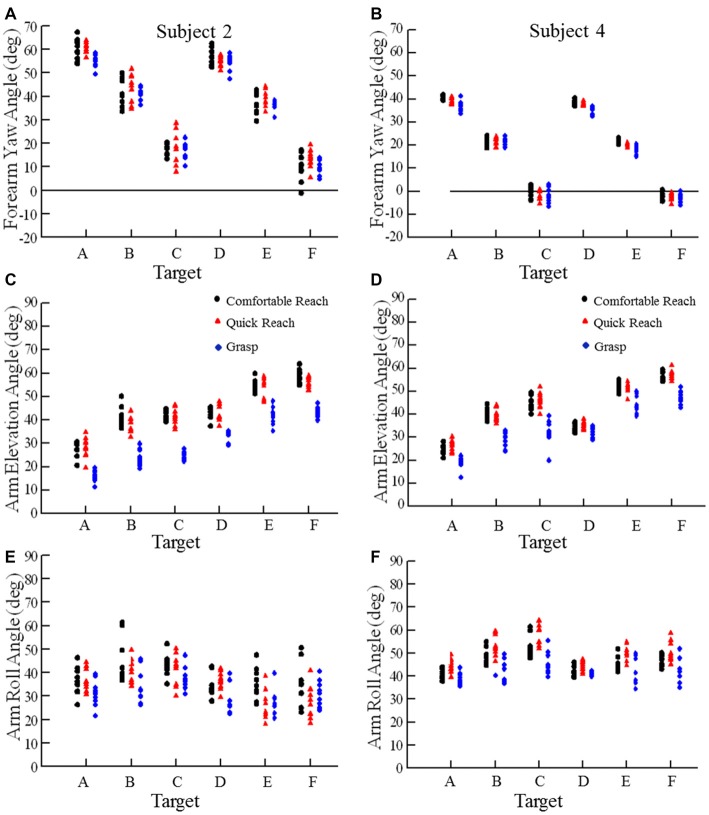
**Examples of forearm yaw, arm elevation, and arm roll angles at movement endpoint for each movement condition.** The targets (A–F) were reached from five different starting positions. Each plotted point is the final forearm yaw angle, arm elevation angle, or arm roll angle for one movement to a specific target with different symbols for comfortable reach (black circle), quick reach (red triangle) and grasp (blue diamond) conditions. Data from subject 2 is on the left **(A,C,E)** and subject 4 on the right **(B,D,F)**.

**Figure 4:**
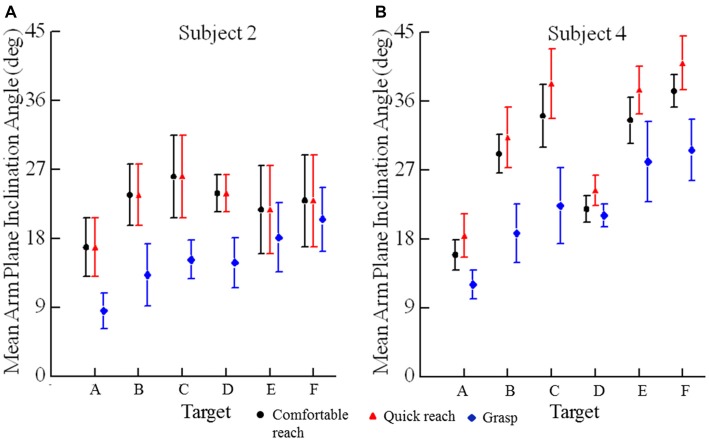
**Mean arm plane inclination angles vs. target for subjects 2 (A) and 4 (B).** Each plotted point is the average of 10 reaches to a single target (A–F), two movements from each of five different starting positions in each of the three conditions. Error bars are ±1 SD.

Recalling the purpose of our study, we asked whether pointing or grasping movements starting from different locations would exhibit low variability in arm posture at the endpoint. Examination of the arm elevation and roll angles and forearm yaw angles for movements from the widely spaced starting hand positions indicated low variability in arm orientation at endpoint for each target across the three reaching conditions and the 5–7 starting positions. Data from two subjects are shown in Figures 3, 4, one with relatively large variations (subject 2) and the other with small variations (subject 4). Figure 3 shows the variation of forearm yaw, arm elevation and arm roll at the endpoint of movements to the six targets under the three different reaching conditions. Within a single reaching condition the range of final segment angles was usually 10° or less, although for some targets in some subjects the range of final segment angles was up to 20° (e.g., Figure 3E). The same data can be expressed in terms of the arm inclination angle, derived from the humeral elevation θ and roll ζ angles as explained in the methods. As can be seen in Figure 4 the variations of arm inclination angle are relatively small for each target. It can be inferred from these measurements that the arm’s posture at a given target is rather similar regardless of the start position, albeit with some variability as detailed below.

**Figure 5:**
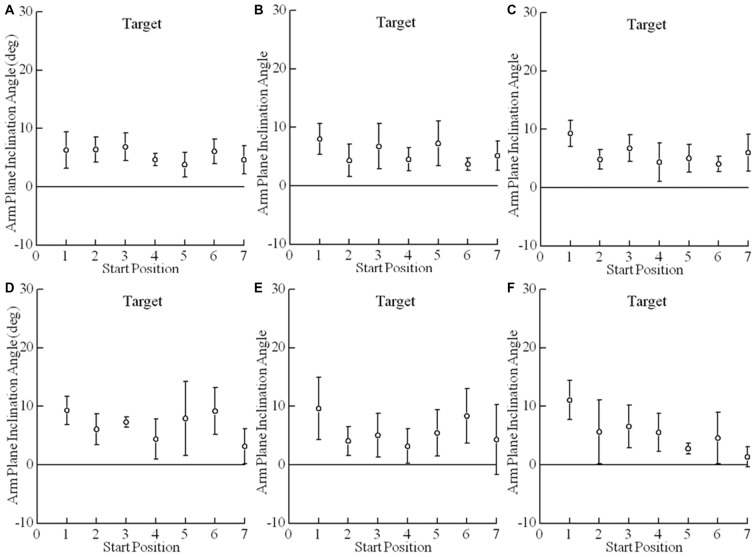
**Arm posture at a given target is essentially independent of the arm’s start position.** Shown here are the mean arm plane inclination angles vs. start positions for comfortable and fast speed reaches for subject 6. Each plotted point is the average of four reaches (2 comfortable speed reaches and 2 fast speed reaches) to a single target **(A–F)** from a single start position **(1–7)**. Error bars are ±1 S.D. Note that the variability of arm posture for repeated movements to a given target is commensurate with the variability across start positions.

**Figure 6:**
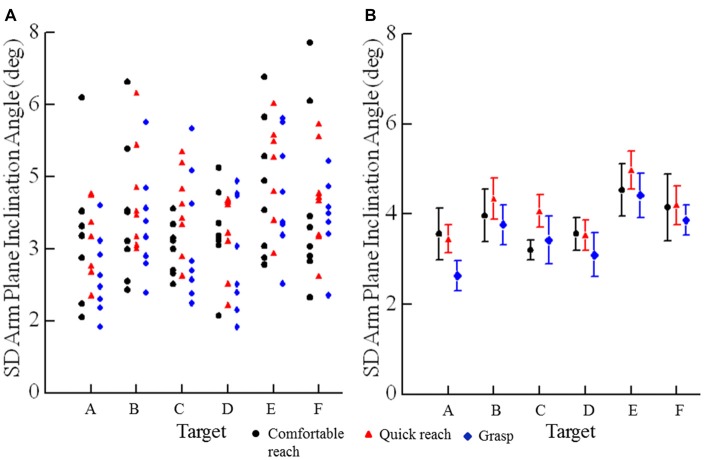
**Variability of angles of arm plane inclination for movements in the three conditions: comfortable reach, quick reach, and grasp. (A)** Each plotted point is an individual subjects’ standard deviation of the angle of inclination for all movements to a specific target (A–F) under a specific condition (comfortable reach, quick reach, or grasp). **(B)** Each plotted point is the mean of standard deviations for eight subjects of the angle of inclination for a specific target under a specific condition. Error bars are ±1 SE.

Trial-to-trial variability of final arm postures was measured as the standard deviation of the arm plane inclination angles for movements from all starting positions to a target for a given reaching condition. The angle of inclination of the arm plane exhibited low variability at each target. For example, data from one subject at each target for comfortable and fast reaches from the seven different starting positions shows that the mean arm plane inclination angle had low variation for individual starting positions and a small range of mean final angles across start positions (Figure 5). Note that the variability of arm posture for repeated movements to a given target from one start position is commensurate with the variability to a given target across start positions. Averaged across all eight subjects the variability of the arm plane inclination was less than 5° at each target (Figure 6B). However, the variability was smaller for targets on the left side (below 3.5° on average), while the middle targets, along with the lower right target, all had slightly larger variability, above 4.0° on average (main effect of target: *F*_(5,35)_ = 3.73, *p* = 0.011). There were no differences in variability of inclination angles among reaching conditions as shown in Figures 6A,B (main effect of condition: *F*_(2,14)_ = 1.93, *p* = 0.18) and no interaction between target location and reaching condition (*F*_(10,70)_ = 0.61, *p* = 0.8). In most subjects the variability of the inclination angles was between 2–4° for all targets and movement conditions, though higher values up to 7° were also observed (Figure 6A). Importantly, the variability of the final inclination angles for movements from one start position was similar to the variability of movements from the widely different start positions for comfortable and fast reaches. Specifically, the variability of final inclination angles from a single start position to a single target averaged 3.5° across subjects, targets and start positions. By comparison, variability from all start positions to a single target averaged 4.2° across subjects and targets for comfortable and quick reaches. In summary, our results demonstrate that subjects reached to or grasped a given target with a characteristic but slightly varying arm configuration from trial to trial. As we detail further below, the characteristic posture at each target was essentially independent of the arm’s start position, and the variability of the index fingertip position at movement termination.

Starting position for comfortable and quick reaches had only very small effects on final arm orientation (Figures 5, 6; *F*_(4,5)_ = 4.4, *p* = 0.009). Mean final arm inclination angles across targets differed between starting hand positions 2 and 3 (*p* = 0.045) and 2 and 5 (*p* = 0.021). However, in both cases the differences in mean final arm posture averaged only 3° (e.g., Figure 5 — mean arm plane inclination angles differ when moving from start positions 2 and 5 to targets A and F in this subject). This demonstrates that the effects of very large differences in starting hand positions on final arm posture were negligible. In fact, this effect was less than the inherent variability of the arm’s posture on repeated movements to the same target from the same initial position (i.e., SD = 3.5°), as described in the previous paragraph. The fact that there was no difference between targets 3 vs. 5 but there were differences between 2 vs. 5 and 1 vs. 5 suggest that this small statistical effect may be idiosyncratic. This point in reinforced by the fact that in grasping there was a statistical difference only between start positions 3 vs. 4 averaging 3.7° across all targets.

## Discussion

We have demonstrated that the arm’s posture after moving the fingertip to a given spatial location is nearly the same regardless of the arm’s start position. Thus, by and large, a Donders like law is obeyed for pointing movements of the arm, whether made at comfortable speeds or quickly. Similarly, when reaching to grasp an object, the arm’s posture at a given location is nearly the same regardless of the arm’s initial location. The results of our grasping study are in keeping with previous findings (Schot et al., 2010) concerning reaches to grasp spheres, which does away with the confound of object orientation. Schot et al. (2010) reported that the arm’s posture is best predicted by the sphere’s position relative to the subject, rather than its start position. Importantly, we found that the variability of arm configurations for repeated movements to a target from the same start position was comparable to the variability when starting from widely different positions (3.5° vs. 4.2°). This novel finding strongly suggests that most of the variability in posture at a given target is due to movement *per se* and not the result of different starting locations, as has been previously suggested (Soechting et al., 1995; Gielen et al., 1997; Admiraal et al., 2004).

Our study differed in important respects from most past studies on the issue (Straumann et al., 1991; Hore et al., 1992; Miller et al., 1992; Medendorp et al., 2000). Our subjects moved within a large and natural workspace. The arm was not restricted to being fully extended or otherwise constrained and the subjects, although seated, were free to move as they wished. They were given explicit instructions as to what to point to and how, an issue that will be dealt with further below. In reference to the title of our article, the signal is that the arm obeys a Donders like law. Its posture at a given location moved to is essentially the same and independent of the arm’s start position, excepting some idiosyncrasies as noted in the results. The noise is the variability of this posture from trial to trial. Most of this variability is inherent to movement *per se* and it is small with respect to the full range of possible orientations (up to 180° for the arm plane). On the latter point our results are in full agreement with all past studies of the issue except the one by Soechting et al. (1995), which has been the most influential in rejecting a Donders like law for the arm. Possible reasons for the discrepancy are given further below.

### A Practical Perspective

In its essentials, what we have demonstrated quantitatively can be readily appreciated in a classroom demonstration. Place an object on a table and ask a subject to make a pointing movement to it, specifying what part of the object must be pointed to. One will appreciate that the subject’s arm adopts a characteristic posture when pointing to the object, regardless of where the arm was initially located at movement onset. One can also take photographs of the arm once the target is reached. Subtraction of the consecutive images will result in a black image, demonstrating that the final posture of the arm was essentially similar from trial to trial. The Donders like rule of the arm can also be demonstrated on oneself. Place yourself in front of a mirror and point to the tip of your nose from any number of starting arm positions. You will observe in the mirror that your arm’s posture is highly similar from trial to trial. Thus, contrary to the impression one may have in reading the literature on the subject there is an obvious signal, as stated by Admiraal et al. (2004) “… arm postures are quite consistent and reproducible within and across participants”. In the remainder of the discussion we provide a retrospective and detailed comparison of our results with previous studies, propose two potential sources contributing to variability and relate our findings to the basic mode of operation of the motor cortex that we have hypothesized (Capaday et al., 2013).

### Retrospective Comparison with Other Studies

In our study, the trunk, wrist and index finger were free to move and such movements, among other sources of variability, can lead to slightly different arm postures at the same location. Consequently, some variability in limb posture at a given location is to be expected. Nonetheless, given the unconstrained nature of our task it is remarkable that the variability of the arm’s orientation was on average only ~4° at any one target. It is equally remarkable that this value is similar to that measured (~2.8–4.8°) in studies of pointing with the fully outstretched arm (Hore et al., 1992; Miller et al., 1992), a task that substantially reduces the number of available degrees of freedom. The variability of arm posture we report is also quite similar to that reported (3–4°) by Gielen et al. (1997) who braced the wrist and fingers. Reflecting on saccadic eye movements, Helmholtz commented “Accordingly, we must not expect quite the same precision in the eye as in a scientific instrument, although under ordinary conditions normal eyes do obey the laws of Donders and Listing pretty accurately.” (quotation in Alpern, 1969). The arm has far more degrees of kinematic freedom than the eye and it is attached to the body, which has multiple degrees of additional freedom. Consequently, we should not expect the variability of the arm’s posture to be commensurate to that of the eye’s (~0.5–1.9° in humans), nor draw conclusions on arm movements based on the behavior of the eye. Gielen et al. (1997) acknowledged that “The fact that previous studies overlooked violations of Donders’ law is not surprising in the light of the results in this study that violations of Donders’ law are typically rather small, namely a few degrees”, but dismiss it as a basic kinematic rule. The consistent rejection of a Donders’ like law (e.g., Miller et al., 1992; Gielen et al., 1997; Admiraal et al., 2004) on the basis of admittedly small deviations is arbitrary and more importantly detrimental to investigations of central neural mechanisms underlying kinematic rules. As we find a negligible effect of widely different arm start positions on the final posture at a given target and patently similar postures, we find no basis for rejecting a Donders’ like law for the arm, as defined in the introduction.

We have mentioned the problem of instructions as to what to point to and how. Of the past studies on Donders’ law as it may apply to the arm, the one by Soechting et al. (1995) is most similar to ours, yet our findings and conclusions are contrary. In their study, the range of the arm’s plane of inclination often exceeded 30° at four of the five target locations. By contrast, the range of values we measured at any one target never exceeded 22° in any movement condition and was less than 10° for most subjects. There is thus a considerable discrepancy between our measurements and those of other groups taken together (e.g., Gielen et al., 1997; Admiraal et al., 2004; Hermens and Gielen, 2004) vs. those of Soechting et al. (1995). One possible explanation, is that in their study subjects were instructed to move their arm to touch the tip of a pointer with a pen shaped stylus held in their hand. This leaves considerable freedom as to how to orient the hand held stylus relative to the target pointer tip and it is possible that the stylus was not always held in the same way from trial to trial. In our task, we specifically addressed this sort of ambiguity as subjects were asked to place the pad of their index finger slightly over the marked center of a cylinder i.e., the spatial coordinate was made explicit and the subjects were not required to handle an external object. There was consequently no ambiguity as to the spatial location to which to bring the arm and importantly the subjects could adopt a multitude of possible postures at each target, but they did not. The postures at a given location are not haphazard, they are very closely related. Subjects may vary wrist, finger and trunk orientation, leading to some variability of the arm’s posture. This is true for repeated movements from one target to another, as well as from different start positions to the same target. We stress again that the variability was quite similar in each case. Note also, that Soechting et al. (1995) reported that variability was least for target 7 (30° below shoulder, approx 53 cm from shoulder) and most at target 5 (53 cm from shoulder pointing to midline). Our subject’s workspace is similar to that of Soechting et al. (1995) study, yet for comparable targets (their target 5 vs. our target E, their target 7 vs. our target A) we do not obtain these results. Consequently the choice of start and final positions in our study do not explain the discrepancy.

What might be the reason for the similarity of the arm’s posture at a given spatial location? Khatib et al. (2009) showed that for reaches to a given spatial location the arm’s posture was within a few degrees of that which minimizes muscle effort to maintain the posture. Here we have shown that the arm’s posture at a given reached target is nearly the same independent of its starting position. Significantly, the variability of the arm’s configuration measured by Khatib’s group (De Sapio et al., 2006; Khatib et al., 2009) had a range of some 10°, comparable to what we have measured. These findings contrast with the suggestion of Soechting et al. (1995) that “the final posture minimizes the amount of work that must be done to transport the arm from the starting location”. The work of Khatib and colleagues suggests instead that it is the effort to maintain the arm in the final posture which is minimized. Moreover, the arm configuration that minimizes muscle effort to maintain the final posture can vary over a wider range for some hand positions than others, perhaps explaining the larger variations at some targets than others. Also, the methods used by Khatib et al. (2009) are based on individual subject anthropometry, thereby predicting different postures for different subjects which may be consistent with the variability of individual subject postures at a given target that we and others have observed (e.g., Soechting et al., 1995; Gielen et al., 1997; Admiraal et al., 2004). In any case, we suggest that the similarity of the arm’s posture at a given spatial location is related to the operational mode of the motor cortex, as detailed in the following section.

### The Motor Cortex and Control of the Arm

Our study was motivated, in part, by the need to relate motor cortical function to biomechanical principles of human voluntary movements. We have recently suggested that the basic mode of operation of the motor cortex is to associate a given spatial location with a given arm posture (Capaday et al., 2013). Our observation that the posture of the human arm at a given spatial location is nearly the same regardless of the arm’s starting position is consistent with this hypothesis, as is the finding that in monkeys the discharge of motor cortex neurons is significantly related to the posture attained by the arm at the end of freely made spontaneous movements (Aflalo and Graziano, 2006). Furthermore, microstimulation at the recording point evoked arm postures that matched the postures to which the neurons at that point were best tuned. Related studies have shown that microstimulation of a given cortical point evokes a reproducible muscle activation pattern that is independent of initial arm position and which moves the hand to a common end point with a similar arm posture (Ethier et al., 2006; Graziano, 2006; Van Acker et al., 2013; Griffin et al., 2014). We thus have a principled basis to further our investigations of motor cortical function. Three main questions arise: (1) how is the cortical point(s) that can drive the arm to the intended final position selected; (2) what underlies the variability of the arm’s final posture; and (3) how are the initial postures of the arm and of the body as a whole taken into account in the selection process. In answer to the second question and in relation to the third, we suggest two main sources of variability. That inherent to central neural activity and that due to moment to moment changes in body posture as a whole.

## Conclusions and Epilog

Taken together our findings and aforesaid considerations suggest that a Donders’ like law for the arm should be stated in three parts, as follows. At each spatial location relative to the body to which one points, there corresponds closely related postures of the arm independent of movement speed, or starting arm position. At each spatial location relative to the body at which an object with a given orientation is grasped, there corresponds closely related postures of the arm, regardless of the arm’s starting position. Physiological variability is intrinsic to the rule, the postures will vary movement to movement by a few degrees. Hermens and Gielen (2004) tested the predictions of various posture-based and trajectory-based models of motor planning and control. While they claimed that their experimental results differed significantly from the predictions of all models considered, notwithstanding they acknowledged that “Of the models considered, Donders’ law best predicts the experimental data” (Hermens and Gielen, 2004).

The degrees of freedom issue is classically posed as a problem, it should be viewed as a solution. The human arm does indeed have more degrees of freedom than are needed to reach any spatial location within its workspace. This gives us the capacity to move along arbitrary paths from one position to another, avoid obstacles, as well as to orient the hand arbitrarily at the end point. We suggest that the vagaries from movement to movement are due in part to these extra degrees of freedom and that, for reasons we still do not fully understand, one of a range of very closely related postures is selected. We can call this “noise” and this noise-like physiological variability is not due to movement speed as we have shown. The kinematic variability may also reflect the variability of central neural activity (e.g., Churchland et al., 2006). Additionally, the configuration of the whole body is quite likely taken into account in the selection process, adding another source of, in this case, apparent variability. Understanding the nature of the selection process and the sources of its variability should further our understanding of motor cortical function. More importantly, the Donders’ like law we have demonstrated should strongly inform the search for central neural mechanisms of arm movement coordination. In particular, whether the basic mode of operation of the motor cortex is to associate a given spatial location with a given arm posture.

## Author Contributions

The study was conceived by CC and WGD. The experiments were done by SE and SMH. The data was analyzed by CC, WGD, SE and SMH. The manuscript was written by CC, WGD and SE. All authors approved the final draft.

## Conflict of Interest Statement

The authors declare that the research was conducted in the absence of any commercial or financial relationships that could be construed as a potential conflict of interest.

## References

[B1] Admiraal M. A. Kusters M. J. Gielen S. C. (2004). Modeling kinematics and dynamics of human arm movements. Motor Control 8, 312–338. 15322310 10.1123/mcj.8.3.312

[B2] Aflalo T. N. Graziano M. S. (2006). Partial tuning of motor cortex neurons to final posture in a free-moving paradigm. Proc. Natl. Acad. Sci. U S A. 103, 2909–2914. 10.1073/pnas.0511139103 16473936 PMC1413833

[B3] Alpern M. (1969). “Kinematics of the eye,” in The Eye, ed. Davson H. (New York: Academic Press), 13–25.

[B4] Atkeson C. G. Hollerbach J. M. (1985). Kinematic features of unrestrained vertical arm movements. J. Neurosci. 5, 2318–2330. 4031998 10.1523/JNEUROSCI.05-09-02318.1985 PMC6565321

[B5] Bernstein N. A. (1967). The Co-Ordination and Regulation of Movements. Oxford: Pergammon Press.

[B6] Campolo D. Formica D. Guglielmelli E. Keller F. (2010). Kinematic analysis of the human wrist during pointing tasks. Exp. Brain Res. 201, 561–573. 10.1007/s00221-009-2073-1 19916007

[B7] Capaday C. Ethier C. Van Vreeswijk C. Darling W. G. (2013). On the functional organization and operational principles of the motor cortex. Front. Neural. Circuits 7:66. 10.3389/fncir.2013.00066 23616749 PMC3629310

[B8] Churchland M. M. Yu B. M. Ryu S. I. Santhanam G. Shenoy K. V. (2006). Neural variability in premotor cortex provides a signature of motor preparation. J. Neurosci. 26, 3697–3712. 10.1523/jneurosci.3762-05.2006 16597724 PMC6674116

[B9] Cooke J. D. Virji-Babul N. (1995). Reprogramming of muscle activation patterns at the wrist in compensation for elbow reaction torques during planar two-joint arm movements. Exp. Brain Res. 106, 177–180. 10.1007/bf00241366 8542973

[B10] Cruse H. (1986). Constraints for joint angle control of the human arm. Biol. Cybern. 54, 125–132. 10.1007/bf00320483

[B11] Cruse H. Bruwer M. (1987). The human arm as a redundant manipulator: the control of path and joint angles. Biol. Cybern. 57, 137–144. 10.1007/bf00318723 3620542

[B12] Darling W. G. Cooke J. D. (1987). Changes in the variability of movement trajectories with practice. J. Mot. Behav. 19, 291–309. 10.1080/00222895.1987.10735414 14988049

[B13] De Sapio V. Warren J. Khatib O. (2006). “Predicting reaching postures using a kinematically constrained shoulder model,” in Advances in Robot Kinematics, eds. Lenar J. Roth B. (Berlin: Springer), 209–218.

[B14] Desmurget M. Prablanc C. (1997). Postural control of three-dimensional prehension movements. J Neurophysiol. 77, 452–464. 9120586 10.1152/jn.1997.77.1.452

[B15] Donders F. C. (1876). Versuch einer genetischedn. Erklaerung der Augenbewegungen. Pflugers Archiv fur Die Gesamte Physiologie 13, 373–421.

[B16] Elliott D. Hansen S. Grierson L. E. (2009). Optimising speed and energy expenditure in accurate visually directed upper limb movements. Ergonomics 52, 438–447. 10.1080/00140130802707717 19401895

[B17] Ethier C. Brizzi L. Darling W. G. Capaday C. (2006). Linear summation of cat motor cortex outputs. J. Neurosci. 26, 5574–5581. 10.1523/jneurosci.5332-05.2006 16707808 PMC6675291

[B18] Fitts P. M. (1954). The information capacity of the human motor system in controlling the amplitude of movement. J. Exp. Psychol. 47, 381–391. 10.1037/h0055392 13174710

[B19] Gielen C. C. Vrijenhoek E. J. Flash T. Neggers S. F. (1997). Arm position constraints during pointing and reaching in 3-D space. J. Neurophysiol. 78, 660–673. 9307103 10.1152/jn.1997.78.2.660

[B20] Graziano M. (2006). The organization of behavioral repertoire in motor cortex. Annu. Rev. Neurosci. 29 105–134. 10.1146/annurev.neuro.29.051605.112924 16776581

[B21] Grea H. Desmurget M. Prablanc C. (2000). Postural invariance in three-dimensional reaching and grasping movements. Exp. Brain Res. 134, 155–162. 10.1007/s002210000427 11037282

[B22] Griffin D. M. Hudson H. M. Belhaj-Saif A. Cheney P. D. (2014). EMG activation patterns associated with high frequency, long-duration intracortical microstimulation of primary motor cortex. J. Neurosci. 34, 1647–1656. 10.1523/jneurosci.3643-13.2014 24478348 PMC3905140

[B23] Hermens F. Gielen S. (2004). Posture-based or trajectory-based movement planning: a comparison of direct and indirect pointing movements. Exp. Brain Res. 159, 340–348. 10.1007/s00221-004-1959-1 15526192

[B24] Hollerbach M. J. Flash T. (1982). Dynamic interactions between limb segments during planar arm movement. Biol. Cybern. 44, 67–77. 10.1007/bf00353957 7093370

[B25] Hore J. Watts S. Vilis T. (1992). Constraints on arm position when pointing in three dimensions: Donders’ law and the Fick gimbal strategy. J. Neurophysiol. 68, 374–383. 10.1007/bf00227256 1527564

[B26] Khatib O. Demircan E. De Sapio V. Sentis L. Besier T. Delp S. (2009). Robotics-based synthesis of human motion. J. Physiol. Paris 103, 211–219. 10.1016/j.jphysparis.2009.08.004 19665552 PMC2782476

[B27] Kistemaker D. A. Wong J. D. Gribble P. L. (2010). The central nervous system does not minimize energy cost in arm movements. J. Neurophysiol. 104, 2985–2994. 10.1152/jn.00483.2010 20884757

[B28] Liebermann D. G. Biess A. Friedman J. Gielen C. C. Flash T. (2006). Intrinsic joint kinematic planning. I: reassessing the Listing’s law constraint in the control of three-dimensional arm movements. Exp. Brain Res. 171, 139–154. 10.1007/s00221-005-0265-x 16341526

[B29] Medendorp W. P. Crawford J. D. Henriques D. Y. Gisbergen J. A. Gielen C. C. (2000). Kinematic strategies for upper arm-forearm coordination in three dimensions. J. Neurophysiol. 84, 2302–2316. 10.1109/cdc.2008.4739155 11067974

[B30] Miller L. E. Theeuwen M. Gielen C. C. (1992). The control of arm pointing movements in three dimensions. Exp. Brain Res. 90, 415–426. 10.1007/bf00227256 1397156

[B31] Misslisch H. Tweed D. Fetter M. Vilis T. (1994). The influence of gravity on Donders’ law for head movements. Vision Res. 34, 3017–3025. 10.1016/0042-6989(94)90275-5 7975337

[B32] Nakayama K. Balliet R. (1977). Listing’s law, eye position sense and perception of the vertical. Vision Res. 17, 453–457. 10.1016/0042-6989(77)90038-4 878335

[B33] Papaxanthis C. Pozzo T. Schieppati M. (2003). Trajectories of arm pointing movements on the sagittal plane vary with both direction and speed. Exp. Brain Res. 148, 498–503. 12582833 10.1007/s00221-002-1327-y

[B34] Quaia C. Optican L. M. (2003). “Three dimensional rotations of the eye,” in Physiology of the Eye: Clinical Application, eds. Kaufman P. L. Alm A. 10th edition (St. Louis: Mosby).

[B35] Schot W. D. Brenner E. Smeets J. B. (2010). Posture of the arm when grasping spheres to place them elsewhere. Exp. Brain Res. 204, 163–171. 10.1007/s00221-010-2261-z 20567809 PMC2892064

[B36] Soechting J. F. Buneo C. A. Herrmann U. Flanders M. (1995). Moving effortlessly in three dimensions: does Donders’ law apply to arm movement? J. Neurosci. 15, 6271–6280. 7666209 10.1523/JNEUROSCI.15-09-06271.1995 PMC6577688

[B37] Straumann D. Haslwanter T. Hepp-Reymond M. C. Hepp K. (1991). Listing’s law for eye, head and arm movements and their synergistic control. Exp. Brain Res. 86, 209–215. 10.1007/bf00231055 1756791

[B39] Tweed D. Cadera W., Vilis T. (1990). Computing three-dimensional eye position quaternions and eye velocity from search coil signals. Vision Res. 30, 97–110. 10.1016/0042-6989(90)90130-d 2321369

[B38] Tweed D. Vilis T. (1990). Geometric relations of eye position and velocity vectors during saccades. Vision Res. 30, 111–127. 10.1016/0042-6989(90)90131-4 2321357

[B44] Tweed D. Vilis T. (1992). “Listing’s law for gaze-directing head movements,” in The Head-Neck Sensory-Motor System, eds Berthoz A. Graf W. Vidal P. P. (New York, NY: Oxford University Press), 387–391.

[B40] Uno Y. Kawato M. Suzuki R. (1989). Formation and control of optimal trajectory in human multijoint arm movement - minimum torque change model. Biol. Cybern. 61, 89–101. 10.1007/bf00204593 2742921

[B41] Van Acker G. M. Amundsen S. L. Messamore W. G. Zhang H. Y. Luchies C. W. Kovac A. (2013). Effective intracortical microstimulation parameters applied to primary motor cortex for evoking forelimb movements to stable spatial end points. J. Neurophysiol. 110, 1180–1189. 10.1152/jn.00172.2012 23741044 PMC3763095

[B42] Virji-Babul N. Cooke J. D. (1995). Influence of joint interactional effects on the coordination of planar two-joint arm movements. Exp. Brain Res. 103, 451–459. 10.1007/bf00241504 7789451

[B43] Wang X. (1999). Three-dimensional kinematic analysis of influence of hand orientation and joint limits on the control of arm postures and movements. Biol. Cybern. 80, 449–463. 10.1007/s004220050538 10420570

